# Anterior Uveitis in Patients With Methylenetetrahydrofolate Reductase (MTHFR) Gene Polymorphisms: A Case Series

**DOI:** 10.7759/cureus.117191

**Published:** 2026-09-30

**Authors:** Nancy Zakaria, Caroline A Tawfik, Samir Shoughy

**Affiliations:** 1 Ophthalmology, Alexandria University, Alexandria, EGY; 2 Ophthalmology, Ain Shams University, Cairo, EGY; 3 Ophthalmology, Dr. Samir Eye Clinic, Alexandria, EGY

**Keywords:** a1298c, autoimmune disease, c677t, genetic polymorphism, homocysteine, mthfr gene, uveitis

## Abstract

Methylenetetrahydrofolate reductase (*MTHFR*) gene polymorphisms have not, to our knowledge, previously been reported in association with anterior uveitis.

We retrospectively reviewed the medical records of three consecutive female patients with anterior uveitis and documented *MTHFR *gene polymorphisms. All patients presented with unilateral nongranulomatous anterior uveitis, and an extensive laboratory evaluation excluded other recognized causes of uveitis. A 37-year-old woman with recurrent left anterior uveitis had a heterozygous C677T polymorphism. A 33-year-old woman presenting with her first episode of right anterior uveitis had a heterozygous A1298C polymorphism. A 50-year-old woman with recurrent anterior uveitis had compound heterozygous C677T and A1298C polymorphisms.

*MTHFR* gene polymorphisms may be a consideration in selected patients with recurrent unexplained anterior uveitis, particularly when conventional autoimmune testing, including HLA-B27, is negative. Further studies are required to clarify whether this observed association is causal, its clinical implications, and any potential relevance to management.

## Introduction

Autoimmune etiology is driven by a synergistic interaction between genetic predisposition and environmental triggers [1]. Among the metabolic factors implicated in this cascade, dysfunctional folate metabolism and elevated homocysteine (Hcy)--an intermediate in the methionine cycle--play a prominent role. Accumulation of Hcy has been increasingly documented across various autoimmune pathologies, particularly uveitis-associated disorders such as Behçet’s disease (BD), ankylosing spondylitis (AS), and multiple sclerosis (MS) [2].

Methylenetetrahydrofolate reductase (*MTHFR*) is an FAD-dependent flavoprotein that catalyzes the NADPH-dependent reduction of 5,10-methylenetetrahydrofolate to 5-methyltetrahydrofolate. This key step in the folate cycle provides one-carbon donors for critical metabolic processes, including nucleic acid synthesis, amino acid metabolism, and cellular methylation, while regulating Hcy remethylation to methionine alongside methionine synthase and vitamin B12 [3,4]. Encoded on chromosome 1p36.22, *MTHFR* shares a tandemly transcribed promoter with other folate cycle enzymes [5]. The C677T (rs1801133; Ala222Val) and A1298C (rs1801131; Glu429Ala) single-nucleotide polymorphisms are the only variants with confirmed functional impacts. C677T reduces FAD affinity and thermolability, decreasing enzyme activity by ~35% per mutant allele, whereas A1298C reduces activity without independently causing hyperhomocysteinemia (HHcy) [3,5,6]. Together or individually, these variants impair MTHFR function and disrupt folate metabolism. Heterozygous C677T and A1298C *MTHFR* gene polymorphisms are common in the general population.

Impaired remethylation of Hcy to methionine, often driven by *MTHFR* gene polymorphisms, leads to HHcy [7]. The resulting systemic toxicity and endothelial dysfunction are strongly linked to cardiovascular and neurological morbidity [8]. Beyond these classic manifestations, the full clinical impact of Hcy-driven pathology remains incompletely defined. *MTHFR* gene polymorphisms may be a consideration in selected patients with recurrent unexplained anterior uveitis, particularly those with a vaso-occlusive/stroke history.

Here, we report a novel association between *MTHFR* gene polymorphisms and acute anterior uveitis, expanding the known disease spectrum associated with folate cycle disruption.

## Case presentation

The first case was a 37-year-old female who presented with blurry vision in her left eye and a 10-year history of recurrent anterior uveitis in the left eye. Review of systems noted vertigo and vague lower back pain. Baseline best-corrected visual acuity (BCVA) was 20/50 in the right eye and 20/200 in the left eye; intraocular pressure (IOP) was 12 mmHg in both eyes. Ocular examination revealed active non-granulomatous anterior uveitis (4+ AC cells, 3+ flare), an irregular pupil, and a posterior subcapsular cataract in the left eye with a normal fundus in both eyes and normal examination in the right eye. Initial workup showed mild microcytic hypochromic anemia, elevated ESR, microscopic hematuria/pyuria, negative HLA-B27 PCR, negative QuantiFERON-TB Gold, and a normal chest X-ray. She was managed with topical prednisolone acetate (q2h) and cyclopentolate (bid); left eye inflammation resolved within two weeks (0.5+ cells, trace flare). Following three months of quiescence, she experienced recurrence in her right eye, followed by two subsequent relapses over six months at three-month intervals, all resolving on the same regimen.

Subsequently, the patient reported a first-trimester spontaneous abortion. An obstetric hypercoagulability and autoimmune panel (D-dimer, AT-III, Protein C/S, Lupus Anticoagulant, ACL IgG/IgM, ANA, Anti-dsDNA, and Factor V/Prothrombin PCR) was negative. However, real-time PCR identified a heterozygous *MTHFR* C677T variant.

The second case was a 33-year-old female who presented with a 4-day history of blurred vision and photophobia in her right eye, with no prior ocular history. Medical history was notable for hypothyroidism following Hashimoto thyroiditis (managed with L-thyroxine), a sleeve gastrectomy 5 years prior, and a cerebrovascular stroke (CVS) 4 years prior. Her previous neurological hypercoagulability workup was negative except for real-time PCR identifying a heterozygous *MTHFR* A1298C variant. On examination, BCVA was 20/100 in the right eye and 20/20 in the left eye; IOP was 9 mmHg in the right eye and 12 mmHg in the left eye. Slit-lamp examination was normal in her left eye, while her right eye revealed intense ciliary injection, 3+ AC cells, 3+ flare, and posterior synechiae resulting in an irregular pupil. Fundus examination was normal in both eyes, though limited by photophobia in her right eye. She was thus diagnosed with acute non-granulomatous anterior uveitis in her right eye, for which she was treated with topical hourly prednisolone acetate and cyclopentolate eye drops. At the 10-day follow-up, AC inflammation improved to 0.5+ cells and trace flare. Complete quiescence was achieved by one month and maintained through her four-month follow-up.

The third case was a 50-year-old female who presented for a routine examination with a history of recurrent, alternating anterior uveitis managed with methotrexate (MTX) 10 mg/week, and a CVS 3 years prior. PCR testing revealed compound heterozygous *MTHFR* variants (C677T and A1298C). Her initial BCVA was 20/32 in the right eye and 20/40 in the left eye; IOP was 11 mmHg in both eyes. Slit-lamp exam showed quiet anterior chambers (0 cells, 0 flare), irregular pupils, and clear lenses, with normal fundi in both eyes, thereby confirming the diagnosis of inactive bilateral non-granulomatous anterior uveitis. Workup revealed mild microcytic hypochromic anemia with anisopoikilocytosis and positive HLA-B27 PCR, with normal CRP, negative QuantiFERON-TB Gold, normal chest CT, and normal sacroiliac joint MRI. Rheumatology evaluation excluded axial spondyloarthritis (axSpA).

MTX was continued, maintaining quiescence for three years until she presented with right eye pain and photophobia. Examination revealed active non-granulomatous anterior uveitis (2+ cells, 1+ flare) in her right eye. She was treated with topical prednisolone acetate and cyclopentolate, and the inflammation resolved within 1 week (0 cells, trace flare).

All clinical data pertaining to the case series are summarized in Table 1.

**Table 1:** Clinical summary of the three cases

Feature	Case 1	Case 2	Case 3
Age	37 years	33 years	50 years
Sex	Female	Female	Female
Uveitis pattern	Recurrent, initially unilateral then alternating	First episode, unilateral	Recurrent, alternating bilateral
Active eye at presentation	Left eye	Right eye	No active inflammation
Active-cell grade at presentation	4+	3+	0
Flare grade at presentation	3+	3+	0
*MTHFR* genotype	Heterozygous C677T	Heterozygous A1298C	Compound heterozygous C677T/A1298C
Cerebrovascular history	None reported	Stroke 4 years earlier	Stroke 3 years earlier
Obstetric history	First-trimester spontaneous abortion	Not reported	Not reported
Relevant associated findings	Microcytic hypochromic anemia, raised ESR, microscopic hematuria/pyuria, posterior subcapsular cataract	Hypothyroidism, previous sleeve gastrectomy, posterior synechiae	HLA-B27 positivity, microcytic hypochromic anemia with anisopoikilocytosis
Systemic therapy	None	None	Methotrexate 10 mg/week
Clinical course	Recurrent relapses at approximately 3-month intervals	Complete remission maintained for 4 months	Quiescence maintained for 3 years on methotrexate before recurrence
Response to topical corticosteroid/cycloplegic therapy	Resolution within 2 weeks	Complete quiescence by 1 month	Resolution within 1 week after recurrence

## Discussion

*MTHFR* gene polymorphisms are well-documented susceptibility factors across multiple autoimmune and inflammatory conditions [2,9]. Notably, a comprehensive meta-analysis confirmed *MTHFR* C677T as a risk factor for BD, MS, and AS, with A1298C also conferring an MS risk [2]. Furthermore, *MTHFR* gene variants are implicated in ocular pathologies such as glaucoma and retinal vein occlusion [10,11]. 

While HLA-B27 remains the primary risk factor for AS, non-MHC regions, including IL4 and MTHFR, are increasingly recognized in its genetic architecture [9,12]. *MTHFR *gene variants may drive pathology through hypercoagulability, where intravascular thrombosis disrupts microvascular perfusion to bone, potentially leading to ischemic tissue damage and osteonecrosis [13].

Mechanistically, HHcy triggers autoimmunity through neoantigen formation, HLA-B27 modification, and induction of Hcy-specific cytotoxic T lymphocytes [14]. The synergistic burden of HLA-B27 and *MTHFR* gene variants likely explains the heightened inflammation in the third case relative to the milder, HLA-B27-negative case in the first case. Hcy further amplifies intraocular inflammation (IOI) via NF-κB activation and upregulation of uveitogenic cytokines (TNF-α, IL-1β, IL-6, IL-8, IL-12) [14-17].

Given that MTHFRdrives cofactor-dependent Hcy remethylation and secondary folate depletion, whether nutritional or drug-induced (e.g., MTX, pyrimethamine, trimethoprim or pemetrexed, anticonvulsants, sulfasalazine, metformin, triamterene and alcohol, and oral contraceptives) can aggravate underlying MTHFR dysfunction, creating a compound risk for recurrent uveitis (Figure 1) [18].

**Figure 1:**
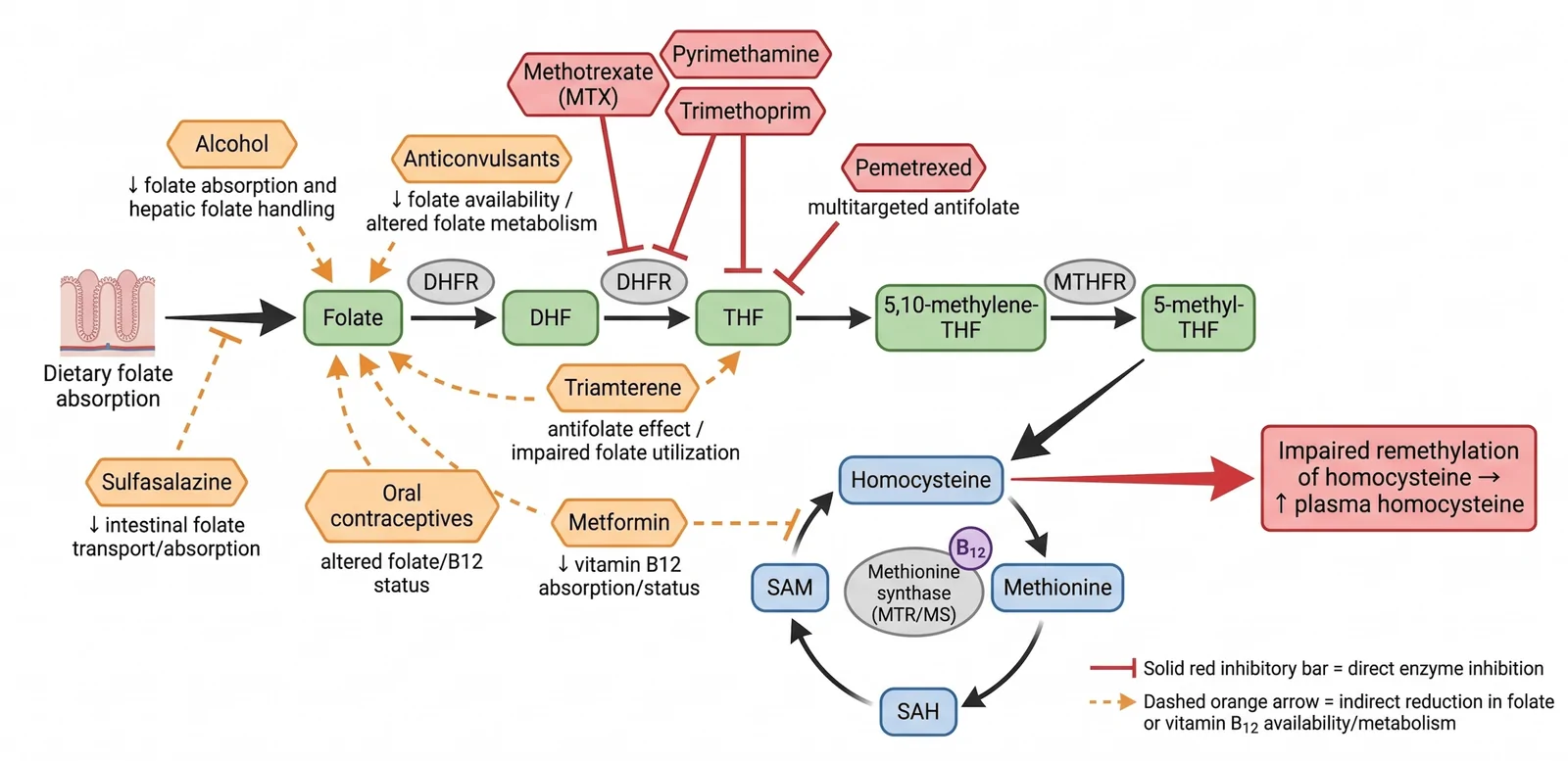
Drug-associated disruption of folate-dependent homocysteine remethylation B12, vitamin B12; DHF, dihydrofolate; DHFR, dihydrofolate reductase; MTHFR, methylenetetrahydrofolate reductase; MTR/MS, methionine synthase/methionine synthase reductase; MTX, methotrexate; SAM, S-adenosylmethionine; SAH, S-adenosylhomocysteine; THF, tetrahydrofolate. Solid red inhibitory bars indicate direct enzyme inhibition; dashed orange arrows indicate indirect reductions in folate or vitamin B12 availability or metabolism. Image created on BioRender.com.

The first case presented with several signs suggestive of an autoimmune process, including recurrent anterior uveitis and spontaneous abortion. The second case experienced only a single episode of uveitis but also evidence of an autoimmune process (hypothyroidism following Hashimoto thyroiditis), as well as a cerebral infarct at the age of 29 years. Moreover, sleeve gastrectomy operation can affect vitamin B12 absorption, which further contributes to HHcy [19]. Whilst the third case was on MTX therapy, which contributes to the state of HHcy owing to its antifolate effect, which likely contributed to her CVS. The *MTHFR* variant in this case may be just an incidental finding rather than a contributing factor alongside HLA-B27. However, rheumatology evaluation excluded axSpA. Other immune-mediated diseases, including reactive arthritis, were carefully excluded in all patients.

*MTHFR* gene testing was performed before the diagnosis of uveitis. *MTHFR *gene testing is not routinely ordered in cases of anterior uveitis. In our patients, other causes were carefully excluded. Larger cohort studies and extended monitoring are required to establish *MTHFR* gene polymorphisms as independent risk factors for anterior uveitis. Observing classical HLA-B27 recurrent inflammation in HLA-B27-negative patients reinforces the clinical evidence of non-HLA genetic susceptibility, emphasizing the need to explore novel metabolic and genetic biomarkers in unexplained uveitis.

## Conclusions

In summary, these cases highlight a potential association between *MTHFR *gene polymorphisms, HHcy, and idiopathic recurrent anterior uveitis. Given the small sample size, these observations should be considered hypothesis-generating and warrant further investigation in larger, prospective cohorts to determine whether screening for *MTHFR* gene variants or serum Hcy levels has diagnostic or therapeutic utility, particularly in patients with idiopathic recurrent anterior uveitis, particularly in HLA-B27-negative cases or those with a history of vaso-occlusive events. While this association highlights a potential metabolic driver of IOI, our conclusions are limited by a retrospective design, small sample size, and unmeasured baseline Hcy levels. Prospective, larger-scale studies are warranted to support our hypothesis and to clarify the mechanistic role and therapeutic implications of *MTHFR *gene variants in uveitis.
